# Supplementary material for: Understanding the Synergy of NKp46 and Co-Activating Signals in Various NK Cell Subpopulations: Paving the Way for More Successful NK-Cell-Based Immunotherapy
Source: Cells. 2020 Mar 19;9(3):753. doi: 10.3390/cells9030753 (PMC7140651; doi:10.3390/cells9030753)
Supplement: Supplementary file 1 [file cells-09-00753-s001.pdf]

Supporting Figures for

Understanding the Synergy of NKp46 and Co-Activating Signals in Various NK Cell Subpopulations: Paving the Way for More Successful NK-Cell-Based Immunotherapy

Loris Zamai[1,2], *Genny Del Zotto[3], * Flavia Buccella[1], Sara Gabrielli[1], Barbara Canonico[1], Marco Artico[4], Claudio Ortolani[1] and Stefano Papa[1]

[1] Department of Biomolecular Sciences, University of Urbino "Carlo Bo"; Urbino, Italy;
[2] INFN-Gran Sasso National Laboratory, Assergi, L'Aquila, Italy.
[3] Area Aggregazione Servizi e Laboratori Diagnostici, IRCCS Istituto Giannina Gaslini, Genoa, Italy;
[4] Department of Sensory Organs, Sapienza University of Rome, Italy;
[*] Tel. : (+39) 0722 304319; fax (+39) 0722 304319; e-mail: loris.zamai@uniurb.it.
[*] equally contributed authors

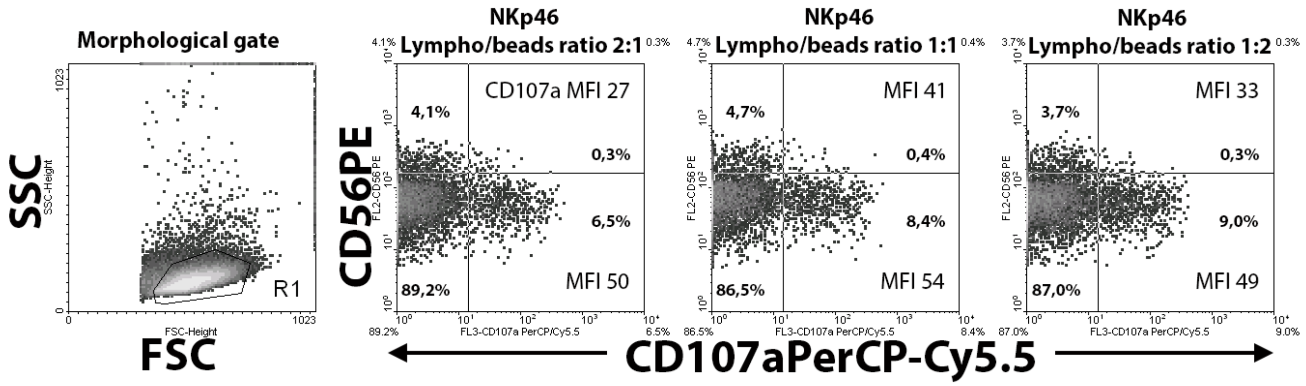

Figure S1. Testing degranulation induced by different ratios of resting lymphocytes and NKp46-coated microbeads. NK population has been selected within the lymphocyte scatter region (R1 in flow cytometric FSC/SSC scatter plot, left panel), gating out dead cells that possess low forward scatter and relatively high side scatter characteristics. Flow cytometry dot plot analyses of different lymphocyte/bead ratios (2:1, 1:1; 1:2) were tested using resting PBMC/NK cells and anti-NKp46 loaded microbeads. $CD56^{dim}$ and $CD56^{bright}$ NK populations are distinguished based on CD56 intensity of expression. Relative percentages of degranulating ($CD107a^{+}$) $CD56^{bright}$ and $CD56^{dim}$ NK cells and CD107a MFI are shown.

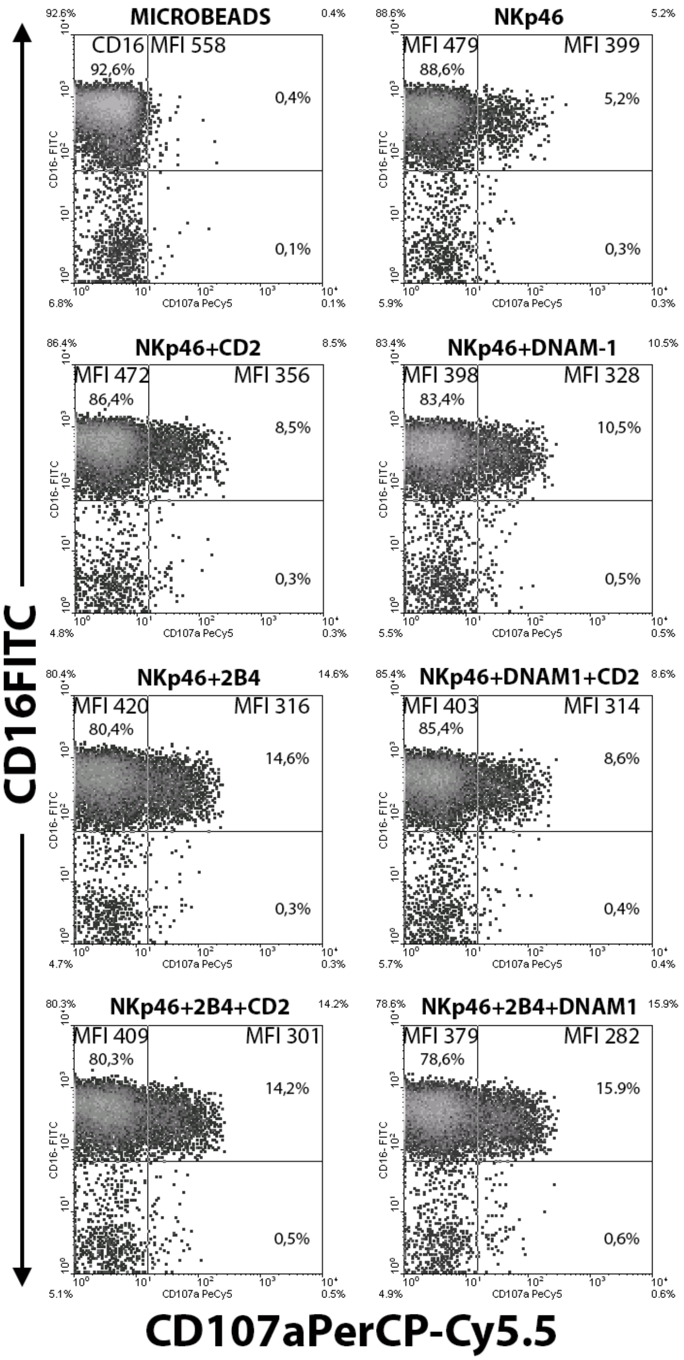

Figure S2. Degranulation of resting NK cells stimulated with different combinations of agonistic mAbs. Flow cytometry dot plot analyses of NK cells. CD16^{bright} (CD3^{neg}CD56^{dim}) and CD16$^{dim/neg}$(CD3^{neg}CD56^{bright}) NK cell subsets are distinguished based on CD16 intensity of expression. Relative percentages of degranulating (CD107a^{+}) CD16^{bright} and CD16$^{dim/neg}$ NK cells and CD16 MFI of CD16^{bright} (either CD107a positive or negative) NK cells are indicated. A partial shedding of CD16 antigen on degranulating (CD107a^{+}) NK cells is evident.

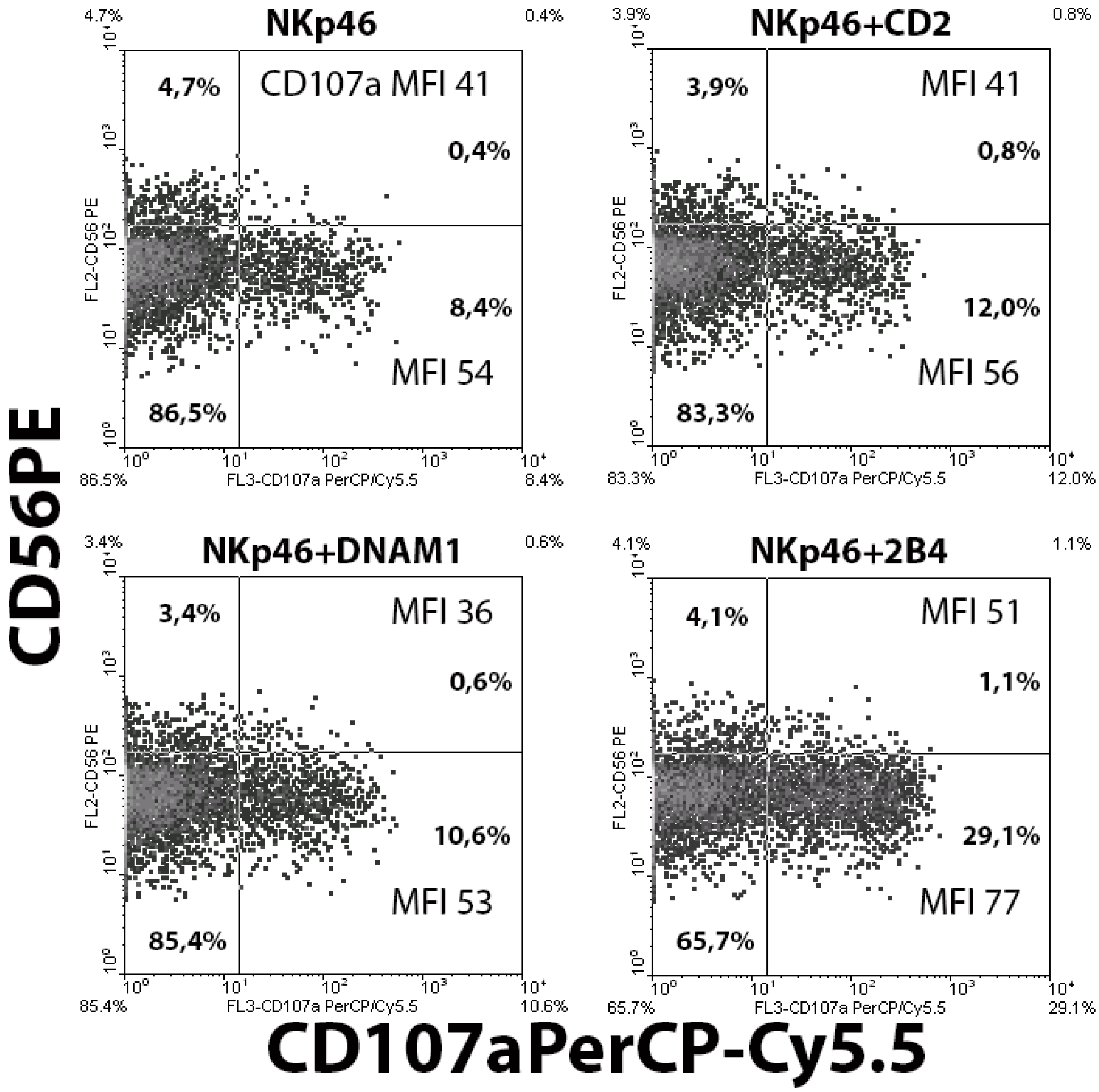

Figure S3. Degranulation of resting CD56^{bright} and CD56^{dim} NK cells stimulated with different combinations of agonistic mAbs. Flow cytometry dot plot analyses of resting CD56^{bright} and CD56^{dim} NK cells. CD56^{bright} (CD3^{neg}CD16$^{dim/neg}$) and CD56^{dim} (CD3^{neg}CD16^{bright}) NK cell subsets are distinguished based on CD56 intensity of expression. CD107a MFI and relative percentages of degranulating (CD107a^{+}) CD56^{bright} and CD56^{dim} NK cells are indicated.

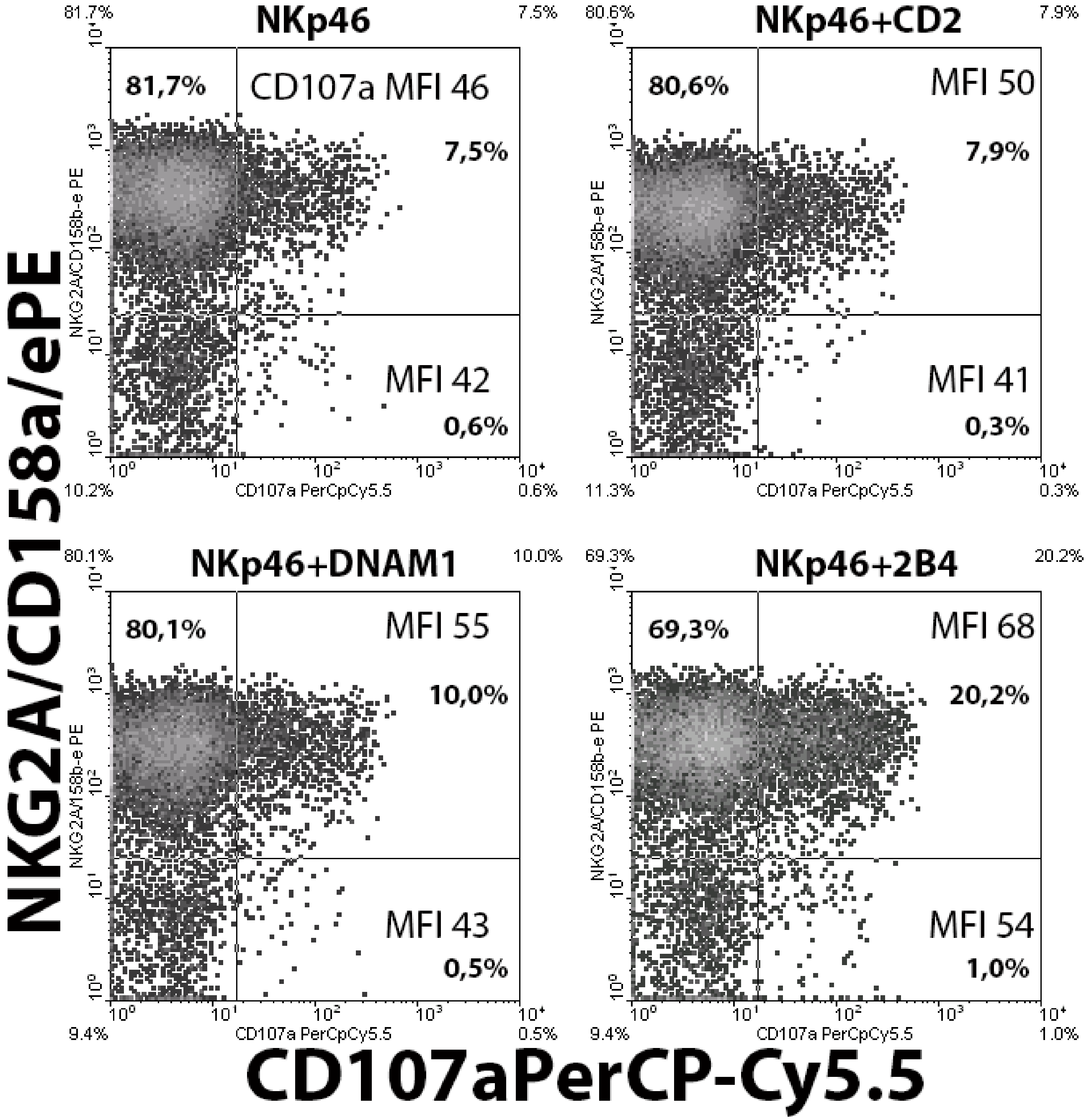

Figure S4. Degranulation of resting licensed and unlicensed CD56^{dim} NK cells stimulated with different combinations of agonistic mAbs. Flow cytometry dot plot analyses of resting licensed and unlicensed CD56^{dim} NK cells. CD56^{dim}CD16^{bright}NKG2A^{+}self-KIR^{+} licensed and CD56^{dim}CD16^{bright}NKG2A^{neg}self-KIR^{neg} unlicensed NK cell subsets are distinguished based on donor HLA class I expression. CD107a MFI and relative percentages of degranulating (CD107a^{+}) licensed and unlicensed CD56^{dim} NK cells are indicated. Donor HLA genotyping was HLA-C1/C1, -Bw4/Bw6.

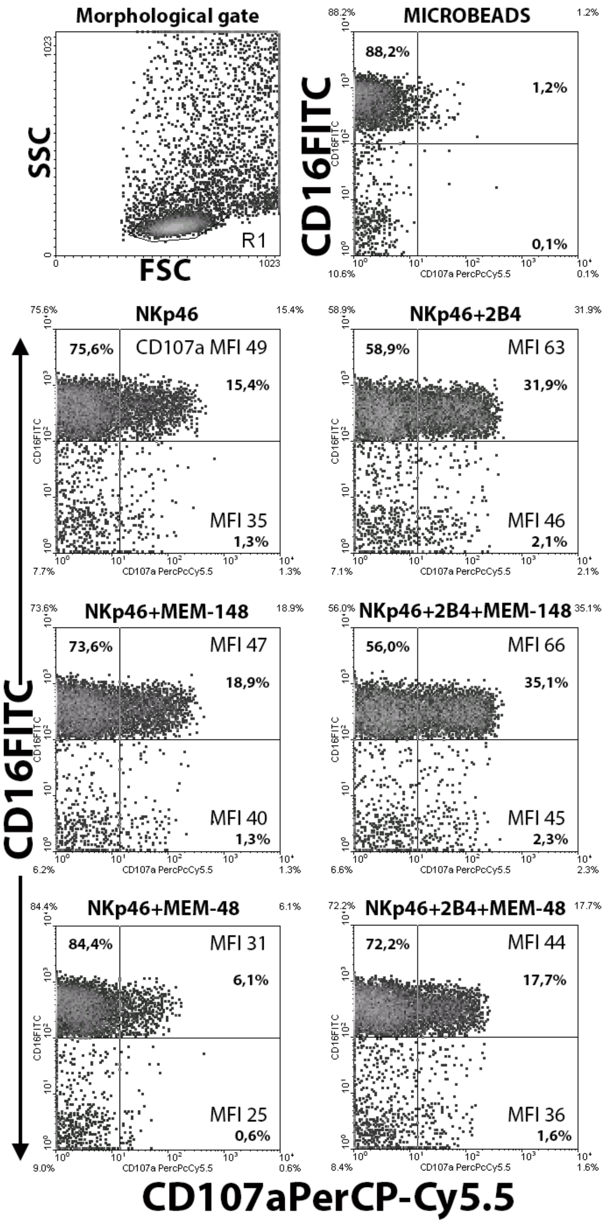

Figure S5. Degranulation of cultured NK cells stimulated with two different anti-LFA-1 β2-chain agonistic mAbs. Flow cytometry dot plot analyses of cultured (one day without cytokine administration) NK cells. NK population has been selected within the lymphocyte scatter region (R1 in flow cytometric FSC/SSC scatter plot, upper left panel), gating out dead cells that possess low forward scatter and relatively high side scatter characteristics. Two different anti-LFA-1 β2-chain (CD18) agonistic mAbs, namely MEM-48 and MEM-148 clones, were tested. $CD16^{bright}$ ($CD3^{neg}CD56^{dim}$) and $CD16^{dim/neg}$($CD3^{neg}CD56^{bright}$) NK cell subsets are distinguished based on CD16 intensity of expression. Relative percentages of degranulating ($CD107a^{+}$) $CD16^{bright}$ and $CD16^{dim/neg}$ NK cells and CD107a MFI are shown.

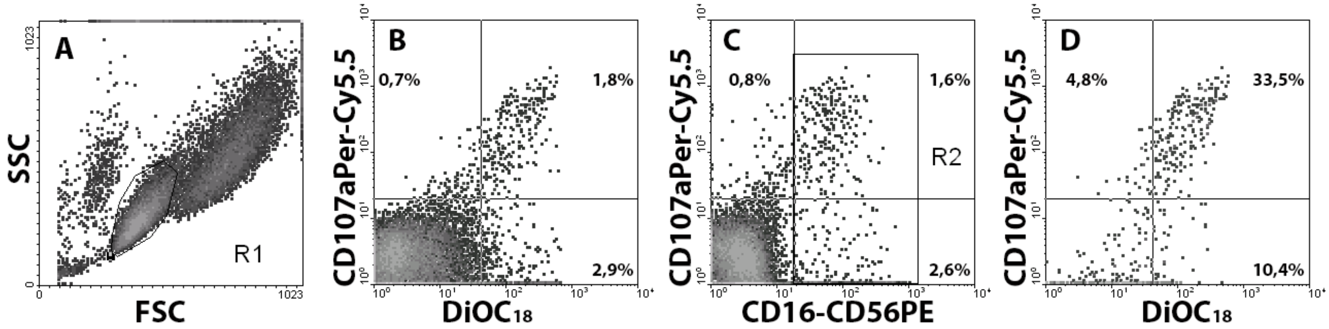

Figure S6. Flow cytometry dot plot analysis of trogocytotic transfer from target to NK cells. Three day IL-2 stimulated PBMCs were incubated (2h) with NK-sensitive green-labelled ($DiOC_{18}$) Jurkat cell line and anti-CD107a PerCP-Cy5.5. (**A**) Lymphocyte population was selected on the basis of flow cytometric scatter (FSC/SSC) characteristics (region **R1**). (**B**) Flow cytometric dot plot shows the correlated expression of $DiOC_{18}$ (green) and CD107a PerCP-Cy5.5. Some lymphocytes (about 5%) acquired $DiOC_{18}$ (green) expression from green-labelled Jurkat cells (trogocytotic transfer) and about 40% of them co-express CD107a antigen. (**C**) NK cells were gated based on CD16-CD56 expression (**R2**). (**D**) Degranulating $CD107a^{+}$ NK cells mostly coexpress $DiOC_{18}$, indicating that $DiOC_{18}$ specifically transfers on NK cells during their degranulation.
